# Transcriptomic Studies of the Effect of *nod* Gene-Inducing Molecules in Rhizobia: Different Weapons, One Purpose

**DOI:** 10.3390/genes9010001

**Published:** 2017-12-21

**Authors:** Irene Jiménez-Guerrero, Sebastián Acosta-Jurado, Pablo del Cerro, Pilar Navarro-Gómez, Francisco Javier López-Baena, Francisco Javier Ollero, José María Vinardell, Francisco Pérez-Montaño

**Affiliations:** 1 Department of Plant Pathology and Microbiology, The Robert H. Smith Faculty of Agriculture, Food and Environment, The Hebrew University of Jerusalem, Rehovot 76100, Israel; ijimgue@us.es; 2 Departamento de Microbiología, Facultad de Biología, Universidad de Sevilla Avda, Reina Mercedes 6, Sevilla 41012, Spain; sacosta@us.es (S.A.-J.); pdelcerro@us.es (P.d.C.); pnavarro2@us.es (P.N.-G.); jlopez@us.es (F.J.L.-B.); fjom@us.es (F.J.O.); jvinar@us.es (J.M.V.)

**Keywords:** RNA-seq, microarray, transcriptome, nodulation, rhizobia, flavonoids, symbiosis

## Abstract

Simultaneous quantification of transcripts of the whole bacterial genome allows the analysis of the global transcriptional response under changing conditions. RNA-seq and microarrays are the most used techniques to measure these transcriptomic changes, and both complement each other in transcriptome profiling. In this review, we exhaustively compiled the symbiosis-related transcriptomic reports (microarrays and RNA sequencing) carried out hitherto in rhizobia. This review is specially focused on transcriptomic changes that takes place when five rhizobial species, *Bradyrhizobium japonicum* (=*diazoefficiens*) USDA 110, *Rhizobium leguminosarum* biovar *viciae* 3841, *Rhizobium tropici* CIAT 899, *Sinorhizobium* (=*Ensifer*) *meliloti* 1021 and *S. fredii* HH103, recognize inducing flavonoids, plant-exuded phenolic compounds that activate the biosynthesis and export of Nod factors (NF) in all analysed rhizobia. Interestingly, our global transcriptomic comparison also indicates that each rhizobial species possesses its own arsenal of molecular weapons accompanying the set of NF in order to establish a successful interaction with host legumes.

## 1. Introduction

Quantification of the global expression level of a bacterial genome in response to specific cues allows the analysis of the transcriptional responses to changing environmental conditions. Several transcriptome profiling methods have been developed in the last years. DNA microarrays and RNA sequencing (RNA-seq) are the two most used genome-wide gene expression quantification techniques [1,2,3,4]. 

Microarrays are based on the hybridization of oligonucleotide DNA sequences representing the entire set of genes of an organism arranged in a grid pattern with DNA molecules representing the transcriptome in a specific condition [5,6,7]. In contrast, in the case of the RNA-seq methodology, the whole complementary DNA (cDNA) is directly and massively sequenced. By mapping the resulting short-sequence reads onto the reference genome, both the relative and absolute expression levels of each gene can be quantified [3,8]. RNA-seq technology does not require species- or transcript-specific probes and, in comparison with microarrays, offers increased specificity and sensitivity. Interestingly, alternative transcripts, small untranslated RNA and transcription start sites can also be identified by using this technique [9,10]. However, the efficiency of RNA-seq is marred with the problem of an overwhelming amount of ribosomal RNA (rRNA) in the data (although rRNA can be almost totally removed during the library preparation), and with variations of reads density along the length of the transcript (normalization of reads density variation along the length of the transcript solves this problem) [11]. For these reasons, the application of multiple transcriptome profiling methods improves the comprehension of the global gene expression picture rather than basing only on one method.

Rhizobia have two distinct life styles, either as free-living soil bacteria or as nitrogen-fixing endosymbionts, called bacteroids, within root nodules of legume host plants. Differentiation from free-living bacteria to bacteroids requires important transcriptomic changes in rhizobia, since bacteria adapt their metabolism to nitrogen fixation and to living inside nodule plant cells. In the case of nodules formed by IRLC-legumes (Inverted Repeat-Lacking Clade), this differentiation process is more drastic and leads to the formation of endoreduplicated bacterial cells that have lost their capacity to divide [12,13]. These bacteroids reduce atmospheric nitrogen to ammonia, which is assimilated by the host plant in exchange of a carbon source and an appropriate environment that promotes the growth of undifferentiated rhizobia [14,15,16]. The oxygen limitation appears to be the key factor driving expression of nitrogen-fixation genes inside root nodules and the *nif* and *fix* cluster of genes are strongly upregulated under microaerobic conditions [17,18,19,20]. However, before this differentiation process occurs, other molecular signals play crucial roles in the phenotypic changes required for the transition between free-living rhizobia and bacteroids. 

The initial steps of the symbiotic interaction between legumes and nitrogen-fixing rhizobia involve reciprocal communication by means of chemical signals produced by both members of the symbiosis. This specific interaction starts when the plant releases to the rhizosphere flavonoids that are recognized by a Lysine transcriptional Regulator (LysR)-type in the bacterium, the NodD protein. NodD induces the expression of the nodulation (*nod*, *nol*, *noe*) genes by binding to specific sequences, the *nod* boxes (NB), located upstream of these genes [21]. These nodulation genes code for enzymes implied in the biosynthesis and export of lipochitooligosaccharides, acylated chitin oligomeric backbones with various functional group substitutions at the terminal or non-terminal residues, also known as Nod factors (NF). These signal molecules are recognized by a specific class of receptor kinases, called Lysin Motif Receptor-Like Kinase (LysM-RLK), which harbour a lysine motif in their extracellular domains. Two LysM receptor kinases (Nod-Factor Receptors, NFR1 and NFR5) are present in roots of different legumes such as the model legumes *Lotus japonicus* and *Medicago truncatula* [22]. Nod factors induce nodule primordia in plant roots and play an essential role in the infection process [23,24,25]. Interestingly, many other symbiotic-related traits are regulated in the first instance via flavonoids and the NodD protein in some rhizobia, such as production of surface polysaccharides, synthesis of phytohormone, motility, quorum sensing, and activation of the type 3 secretion system (T3SS) [26,27,28,29,30,31,32]. However, these symbiotic-related features are not present in all rhizobia, since each microsymbiont has developed its own molecular arsenal to reach the same purpose: the success in the infection of host legumes.

In this review, we extensively searched and analysed the main symbiotic-related transcriptomic studies carried out hitherto in rhizobia by using microarray and/or RNA-seq techniques. Our study is specifically focused on those reports in which it was analysed the global gene expression response of several rhizobial strains to the presence of the key initial symbiotic signals, the inducing flavonoids. The goal of this review is to describe the arsenal of molecular weapons used by each rhizobial strain in the initial steps of its symbiotic interactions with host legumes.

## 2. Rhizobial Transcriptomic Studies Related with Symbiosis

**Figure 1:**
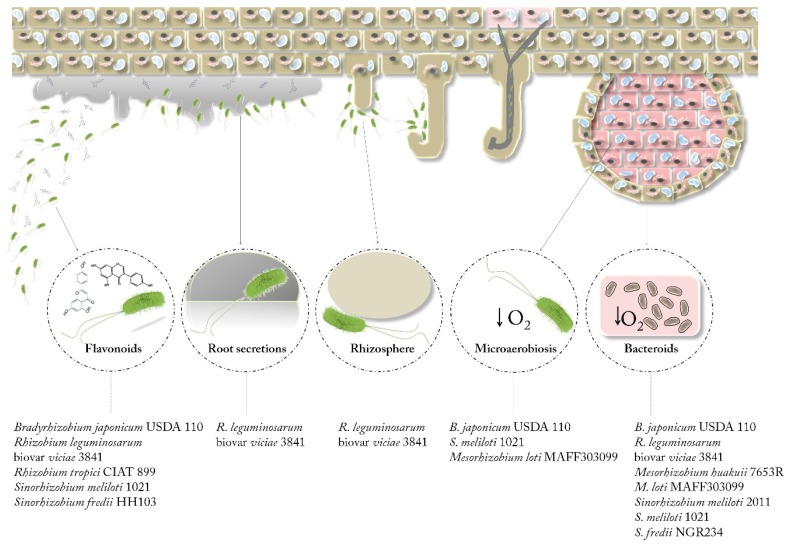
Transcriptomic conditions analysed in this review. Reports compiled in this manuscript were performed in different stages/conditions of the symbiotic process (flavonoids, root secretions, rhizosphere, microaerobiosis and bacteroids).

As previously discussed, the rhizobium-legume interaction is characterized by a complex exchange of signal molecules from both partners that culminates in the formation of nodules, in which biological nitrogen fixation takes place. Two of the most important and studied conditions are the presence of flavonoids (which induce *nod* genes expression) and a microaerobic environment (which triggers the expression of the *nif*/*fix* genes). In fact, the transcriptomic reports published during the last 15 years can be grouped depending on the specific stages of the symbiotic process analysed: bacteroids vs. free-living bacteria, microaerobiosis vs. aerobiosis, and rhizosphere or presence of root exudates or flavonoids vs. non-induced cultures (Figure 1, Table 1).

### 2.1. Bacteroid and Microaerobiosis Conditions

Microarray and/or RNA-seq studies performed in bacteroids or in microaerobiosis conditions has allowed the analysis of the global transcriptomic changes necessary for the reduction of atmospheric nitrogen to ammonia in the symbiosomes of nodules (Table 1). It has been previously described that two Nif and one Fix proteins are the components of the symbiotic nitrogen fixation machinery. The *nif* genes code for the two proteins that constitute the nitrogenase (dinitrogenase and dinitrogenase reductase) as well as for the iron-molybdenum cofactor synthesis proteins, whereas ferredoxins or flavoproteins, the electron supply proteins, are encoded by the *fix* genes [48]. These two main clusters of genes are directly responsible for N_2_ fixation and their expression is controlled primarily by the oxygen tension in rhizobia, although nitrogen availability also affects their expression levels [49,50]. Thus, under low O_2_ tension, the oxygen sensor FixL autophosphorylates, and transfers phosphate to its cognate response regulator FixJ, which in turn up-regulates the transcription of the *nifA* and *fixK* genes. NifA, in conjunction with RpoN (σ^54^ transcriptional factor) activates the transcription of nitrogen fixation genes, such as *nifHDKE* and *fixABCX* operons. Moreover, FixK also induces the transcription of other nitrogen fixation genes, such as the *fixNOQP* and *fixGHIS* operons. 

Interestingly, this hierarchical *fixJ-nifA-fixK* regulatory cascade was fully assessed by performing transcriptomic studies in *Sinorhozobium meliloti* 1021 [39]. The rest of transcriptomic studies confirmed that both *fix* and *nif* clusters are strongly up-regulated in symbiosomes in all analysed rhizobia (Table 1). Interestingly, in *Bradyrhizobium japonicum* and *Mesorhizobium* spp., other groups of genes related with the correct functioning of the nitrogenase enzyme, such as the *hup* (H_2_ uptake), *fdx* (ferredoxin) and *suf* (Fe-S cluster) operons, are commonly activated in symbiosomes [20,33,35]. The transcriptomic studies performed with *rpoN* mutants under low O_2_ concentration conditions indicate that the upregulation of N_2_ fixation-related clusters is mediated by oxygen limitation and the σ^54^ transcriptional factor (RpoN) in *Bradyrhizobium japonicum* USDA 110, *Mesorhizobium huakuii* 7653R and *Sinorhizobium meliloti* 1021 [19,33,35,38]. However, in the case of *M. loti* MAFF303099, the transcriptomic studies performed in parallel under microaerobiosis conditions indicated that most of the up-regulated genes in bacteroids are induced in a NifA-RpoN-independent manner [20]. On the other hand, transcriptomic studies carried out in symbiosomes of *Sinorhizobium* spp. indicate that several processes such as cell division (*ftsZ* gene), DNA, RNA and protein metabolisms are inhibited, which might be correlated with the absence of cell division in fully developed bacteroids [18,37,41]. 

Lastly, the paper published by Roux et al. [40] entails a milestone in the field of transcriptomic research in symbiosis since authors studied for the first time how bacterial and plant gene expression levels vary in different tissues of the indeterminate nodules formed in the symbiosis between *S. meliloti* and *M. truncatula*. 

### 2.2. Rhizosphere and Exudates Conditions

Flavonoids are phenylpropanoid metabolites exuded by plant roots, often in response to elicitors produced by microorganism present in the rhizosphere [51]. These molecules are antimicrobial compounds that inhibit growth of many root pathogens, ranging from bacteria to fungi [52]. However, in rhizobia, flavonoids can act as bacterial growth promoters, chemoattractants, inducers of nodulation genes, determinants of host specificity, and regulators of phytoalexin resistance [53]. Transcriptomic studies performed in rhizosphere conditions or in culture media supplemented with either root exudates or flavonoids have shed light to the repertoire of molecular weapons used by each rhizobial strain in the initial steps of the symbiosis (Table 1). However, only one study has been developed in the case of transcriptomic changes that take place in the rhizosphere and in the presence of root exudates of different host and non-host legumes as well as of non-legume plants. Results obtained by Ramachandran et al. [42] described a common core of rhizosphere-induced genes of *Rhizobium leguminosarum* biovar *viciae* 3841. Despite the fact that most of these genes code for proteins with unknown functions (66%), the up-regulation of well-known genes indicates that several physiological processes are enhanced when this rhizobial species colonizes plant roots, such as acid organic metabolism (*dctA* and *pckA* genes, implied in C4-dicarboxylate transport and Phosphoenolyruvate (PEP)-carboxykinase production, respectively), molecular transport (*rmrA*, coding for a multi-drug resistance family efflux pump) [54] and osmotic adaptation (*ndvA*, whose product is responsible for the export of cyclic β-1-2-glucans to the bacterial periplasm) [55]. The activation of this set of genes appears to be the way *R. leguminosarum* deals with adverse conditions in the plant rhizosphere.

Interestingly, many *Rhizobium leguminosarum* bv. *viciae* (Rlv)3841 genes are induced in the rhizosphere of legume plants, including the *nodABCEFIJLMNO* operon (located on pRL10, the symbiotic plasmid of Rlv3841), but not in the sugar beet rhizosphere. As expected, this *nod* operon is also induced in the presence of pea exudates, containing inducing flavonoids for this strain. Surprisingly, many genes of the non-symbiotic plasmid pRL8 are upregulated only in the rhizosphere of pea, which indicates that pRL8 enables adaptation of *R. leguminosarum* to this specific environment. Among these genes are included those coding for a molybdenum-containing xanthine dehydrogenase-like carbon monoxide dehydrogenase (*cox* cluster) and proteins involved in molybdopterin biosynthesis (*moaA* gene) [42]. However, the exact role of these proteins in the adaptation of *R. leguminosarum* to pea rhizosphere remains to be studied.

## 3. Gene Expression Changes under *nod* Gene-Inducing Molecules: The Arsenal of Weapons

The symbiotic relationship between legumes and nitrogen-fixing rhizobia involves reciprocal communication through chemical signals produced by the plant and the bacterium. The key plant molecules that initiate this communication are flavonoids. It is well known that these inducers activate the expression of the *nod* genes, which code for enzymes involved in the production and export of specific NF that are responsible for root infection and induction of nodule development [53]. However, upregulation of *nod* genes is only the tip of the iceberg in the transcriptomic changes that take place when rhizobia perceive the appropriate flavonoids. Proteomic studies also support this observation since, besides Nod proteins, many other rhizobial proteins are differentially synthetized in the presence of inducing flavonoids [56,57].

### 3.1. Bradyrhizobium Japonicum USDA 110: Diversification in the Activation Pathways for NF Production, Assembly of the T3SS and the Flagellum

*B. japonicum* USDA 110 is an endosymbiont of soybean (*Glycine max*), mung bean (*Vigna radiata*), cowpea (*Vigna unguiculata*) and siratro (*Macroptilium atropurpureum*), among other legumes [58]. In this bacterium, the regulation of symbiotic genes differs from that of other rhizobial strains. Thus, besides NodD1, the two-component regulator NodW, which is phosphorylated by the NodV sensor protein, promotes the transcription of *nod* genes [59,60,61,62,63]. Interestingly, *nodW* seems to play a major role in this strain, since its mutation prevents nodulation in mung bean, cowpea and siratro [59]. In contrast, mutation in *nodD1* causes severe effects in the nodulation with mung bean and siratro, but does not completely block the nodulation process [63]. In both mutants, nodulation with soybean is less efficient, leading to a delay in the formation of nodules [59,60,61,62,63]. Surprisingly, the symbiotic defect of the *nodW* mutant can be suppressed by overexpression of the gene coding for another two-component regulator, *nwsB* [64]. 

**Figure 2:**
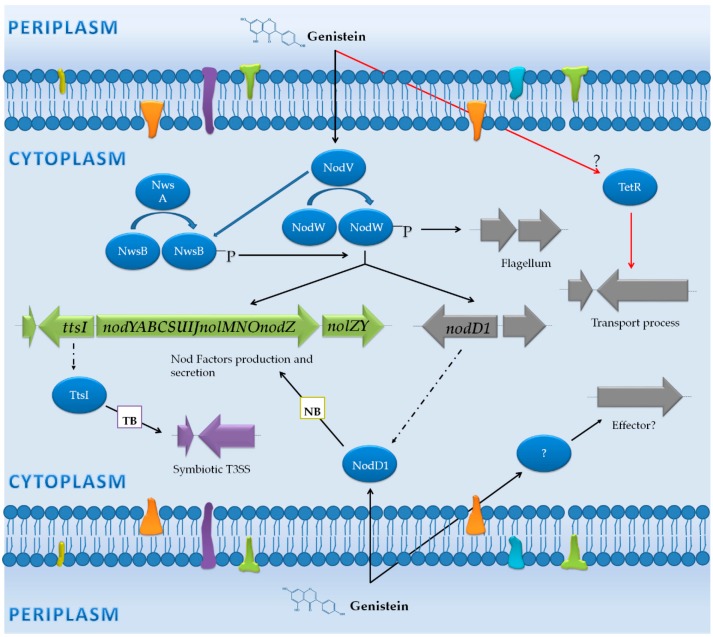
Transcriptomic response in the presence of genistein in *B. japonicum* USDA 110. Proteins are illustrated by blue circles. Protein phosphorylation processes are indicated by blue arrows. The DNA to protein translation process is represented by dotted arrows. Activation and repression of genes are indicated by black and red arrows, respectively. Genes regulated by a *nod* box or a *tts* box are represented in green and purple, respectively. Genes which are not controlled by a *nod* box or a *tts* box are indicated in grey. NB: *nod* box; TB: *tts* box; T3SS: type 3 secretion system.

An analysis of *B. japonicum* USDA 110 transcriptome changes in response to genistein, an inducing flavonoid for this strain, was performed using the wild type strain, a *nodW* mutant and this mutant overexpressing *nwsB* [43]. This work showed that this bacterium responds to genistein by triggering the expression of a battery of 101 genes after 8 h of treatment with this flavonoid (Table S1). Interestingly, three groups of genes differentially expressed were identified: (i) genes controlled by *nod* boxes (NB); (ii) genes regulated by *tts* boxes (TB); and (iii) genes not preceded by a NB or a TB (Figure 2).

#### 3.1.1. Genes Controlled by NB

Among genes induced with genistein, 34 are located within the symbiotic region, being 20 of them dependent on *nod* boxes. As expected, this transcriptomic study demonstrated that NodW is the main regulator in the activation of these 20 genes via NB, despite the fact that the interaction of NodW and NodD1 at NB promoters remains to be clarified. In the absence of NodW, the overexpression of NwsB complements the activation pathway via NB [43]. Previous works described that three sets of genes are located downstream of NB, the *nodYABCSUIJnolMNnodZ* and *nolYZ* operons [65,66] and the *ttsI* gene, the latter coding for the transcriptional activator of T3SS genes [67]. As expected, these genes are strongly up-regulated in a NodW-NwsB dependent manner. In addition, by using sequence comparisons and hybridization experiments, another potential NB has been described in the promoter region of the *bsr1863* gene, which codes for a hypothetical protein of unknown function [68,69]. The expression of this gene is also controlled by NodW and NwsB (Table S1) (Figure 2).

#### 3.1.2. Genes Controlled by TB

The TtsI protein is a transcriptional regulator that binds to conserved sequences located in the promoter region of the T3SS genes, the *tts* boxes (TB). In the transcriptomic study performed by Lang et al. [43], only 6 out of the 35 TB previously described [67,68,69,70] are activated eight hours upon induction with genistein. Thus, the expression of the *gunA2* (*blr1656*), *blr1676*, *bsl18089*, *nopB* (*blr1812*), *pgl* (*blr1993*), and *blr2140* genes is triggered in the presence of flavonoid in a NodW-dependent manner. Although *nopB* is the first gene of an T3SS operon, none of the genes located downstream were activated with genistein in this study, which suggests that probably at the time of sample collection (8 h upon addition of genistein) activation of the transcription of the T3SS genes was only starting. In fact, studies performed previously by using fusions to a reporter gene determined that all T3SS genes are induced with genistein [67]. Furthermore, proteomic studies previously performed also support this fact, since in the presence of genistein different T3SS proteins, such as Blr1649, Blr1806 and GunA2 (Blr1656), are secreted to the extracellular medium [70], although only *gunA2* was detected as activated in the presence of genistein (Table S1) in this transcriptomic study [43].

#### 3.1.3. Genes Not Preceded by a NB or a TB

Most of these genes belong to the flagellar cluster, which could be related to the competitive capacity of the bacterium in the rhizosphere. All these genes are expressed uniformly and depend on NodW. Interestingly, overexpression of *nwsB* is not able to restore expression levels in a *nodW* mutant, which indicates that NwsB complement only genes controlled by NB. Some of the genes that are expressed upon genistein induction code for regulator proteins that could be controlling the expression of the flagellar cluster. Interestingly, Blr6843 and Blr6886 belong to the Multiple antibiotic resistance Regulator (MarR) family of regulators, which are repressor and/or activator proteins that sense environmental changes and may even interact with phenolic compounds [71,72]. 

On the other hand, the differential expression in the presence of genistein of eight genes located outside of the symbiotic region is not due to NodW [43]. Seven of these genes are related to transport processes (*blr2422*, *blr2423*, *bll4319*, *bll4320*, *bll4321*, *bll6621* and *bll6622* genes): Bll6621 and Bll6622 belong to the major facilitator superfamily, while the remaining proteins belong to the resistance-nodulation-division (RND) family. The presence of Tetracyclin Repressor (TetR)-type repressors close to these genes suggests that their expression could be regulated by these repressors [73]. On the other hand, one of the genes that is not regulated by NodW, *blr4684*, codes for an esterase with a patatin type domain present in several pathogen effectors but with unknown function in symbiosis. Interestingly, there are homologues to *blr4684* in other rhizobial strains, such as *Bradyrhizobium* sp. BTAil, *Sinorhizobium fredii* NGR234 and *Mesorhizobium loti* MAFF303099.

Finally, the *nodD1* gene is included in the set of genes not preceded by NB or TB but upregulated by flavonoids and NodW. Interestingly, the expression pattern of *nodD1* is restored by NwsB in the absence of NodW. This expression pattern is also observed in other symbiotic genes, such as *bll1630* (*nolK*), *bll1631* (*noeL*) and *blr2062* (*noeI*) genes, involved in 2-*O*-methyl-fucosylation of NF [61] (Table S1) (Figure 2).

### 3.2. Rhizobium Leguminosarum Biovar Viciae 3841: NF Synthesis and Adaptation to the Rhizosphere Environment

**Figure 3:**
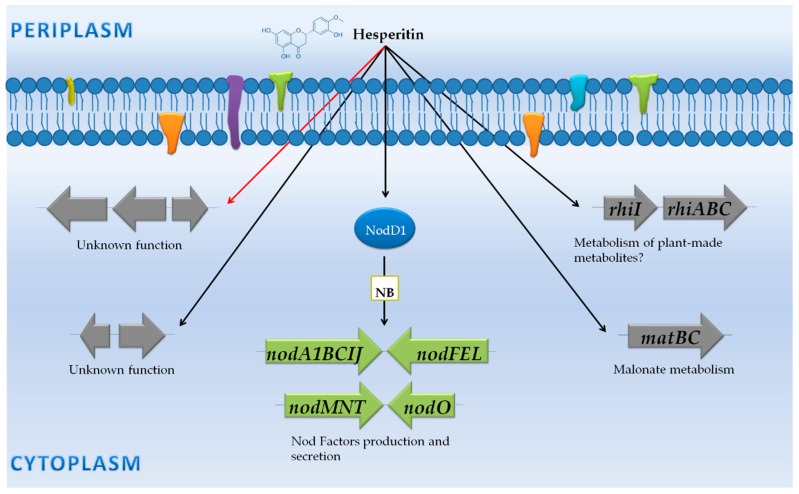
Transcriptomic response in the presence of hesperetin in *R. leguminosarum* bv. *viciae* 3841. Proteins are illustrated by blue circles. Activation and repression of genes are indicated by black and red arrows, respectively. Genes regulated by a *nod* box are represented in green. Genes which are not controlled by a *nod* box are indicated in grey.

*R. leguminosarum* bv. *viciae* 3841 induces the formation of nitrogen-fixing nodules with several hosts, including pea (*Pisum sativum*), lentil (*Lens culinaris*), and *Vicia* spp. [74]. As it is common in rhizobia, the Rlv3841 *nodD* gene, which is transcribed divergently from the *nodABCIJ* operon, is constitutively expressed, although its expression is negatively autoregulated [75,76]. As commented previously, the exhaustive transcriptomic study performed by Ramachandran et al. [42] was focused on the understanding of the adaptation of *R. leguminosarum* bv. *viciae* 3841 to pea, alfalfa and sugar beet rhizospheres in order to establish the sets of genes involved in the general adaptation to the rhizosphere and in the specific adaptation to the pea rhizosphere. In this study, it was also analysed the transcriptomic response to the presence of hesperetin, a *nod* gene-inducing flavonoid of this bacterium. The analysis of these data indicate that 33 genes are differentially expressed (27 upregulated and 6 repressed) upon flavonoid treatment (Table S2), which are distributed between two groups: (i) genes controlled by NB and (ii) genes not preceded by a NB (Figure 3).

#### 3.2.1. Genes Controlled by NB

The presence of at least 5 NB controlling their respective set of *nod* genes had been previously identified in the genome *R. leguminosarum* bv. *viciae* 3841 [77,78,79,80,81,82,83]. As expected, the expression data upon flavonoid induction indicate that the transcriptional units controlled by NB (*nodABCIJ*, *nodFEL*, *nodMNT*, and *nodO*) in this bacterium are strongly upregulated. This set of *nod* genes encoded proteins implied in the synthesis and export of the pool of NF produced by Rlv3841 [42] (Table S2) (Figure 3). 

#### 3.2.2. Genes Not Preceded by a NB

One of the most abundant proteins produced by strains of *Rhizobium leguminosarum* bv. *viciae* in the rhizosphere is RhiA [84]. The *rhiA* gene is part of the *rhiABC* operon that is transcriptionally controlled by the LuxR-type regulator encoded by *rhiR.* It has been previously described that these genes are strongly activated in the rhizosphere of plants via inducing flavonoids. The functions of the proteins encoded by this operon seems to be related with the plant–microbe interaction, possibly by allowing the bacteria to metabolize some plant-made metabolites [85,86]. As expected, the transcriptomic study indicates that the *rhiABC-rhiR* gene cluster is highly activated in the presence of hesperetin. This set of genes is located between nitrogen fixation (*nifHDK*) and nodulation (*nod*) genes on the symbiotic plasmid, pRL10, but are not dependent on a NB. Interestingly, the *matBC* genes are also upregulated in the presence of hesperetin. The *matABC* genes code for a malonyl-CoA decarboxylase, malonyl-CoA synthetase, and a putative malonate transporter, respectively [87,88]. These enzymes are involved in the uptake and conversion of malonate to acetyl-CoA. Again, the upregulation of these genes seems to be orientated towards rhizobial adaptation when the bacterium colonizes the legume root, since the level of free malonate in legumes is known to be increased under symbiotic conditions [89]. Interestingly, in pea roots, the uptake and catabolism of malonate plays an important role in the initial rhizobial attachment and colonization of the root [90]. Thus, transcriptomic data suggest that flavonoid perception and malonate uptake is co-regulated in *R. leguminosarum* bv. *viciae* 3841, probably in order to ensure the attachment to the root surface of a compatible host plant. Lastly, 6 genes, including *flaD*, *lpxC* and *aldA* (encodes for flagellin, *N*-acetylglucosamine deacetylase and alanine dehydrogenase, respectively)*,* and others coding for proteins with unknown functions (RL3912, RL0798, and RL3172), are downregulated in Rlv3841 in the presence of inducing flavonoids but their relevance in symbiosis requires further research (Table S2) (Figure 3).

### 3.3. Rhizobium Tropici CIAT 899: NF Synthesis Also under Stressing Conditions, Production of a Large Variety of NF and Phytohormones

*Rhizobium tropici* CIAT 899 is a broad host-range bacterium that effectively nodulates several legumes, including *Phaseolus vulgaris*, *Leucaena leucocephala*, *Lotus japonicus* and *L. burtii* [91,92,93]. The main characteristics of this strain include its high tolerance to several environmental stresses such as high temperature, acidity or salinity and its capacity to produce a large variety of NF in the presence of inducing flavonoids, such as apigenin [91,94,95]. Surprisingly, under salt stressing conditions, this bacterium induces the synthesis of NF in a NodD1-independent manner [96,97]. Interestingly, genome sequencing of *R. tropici* CIAT 899 revealed the presence of five different *nodD* genes and three different *nodA* genes in the symbiotic plasmid [98]. The *nodA1* gene is located close to *nodD1*, whose encoded protein is the key regulator of NF synthesis upon induction with flavonoids [92,99] and together with *nodBC* comprise the operon responsible for the synthesis of the NF core. The *nodA2* gene, which is adjacent to *nodD2*, is part of a set of genes, including *hsnT* and *nodFE*, implied in unsaturated fatty acid incorporation into NF molecules. Finally, the *nodA3* gene is located downstream the *nodD3* gene but no other symbiotic-related genes have been identified in its vicinity [93].

**Figure 4:**
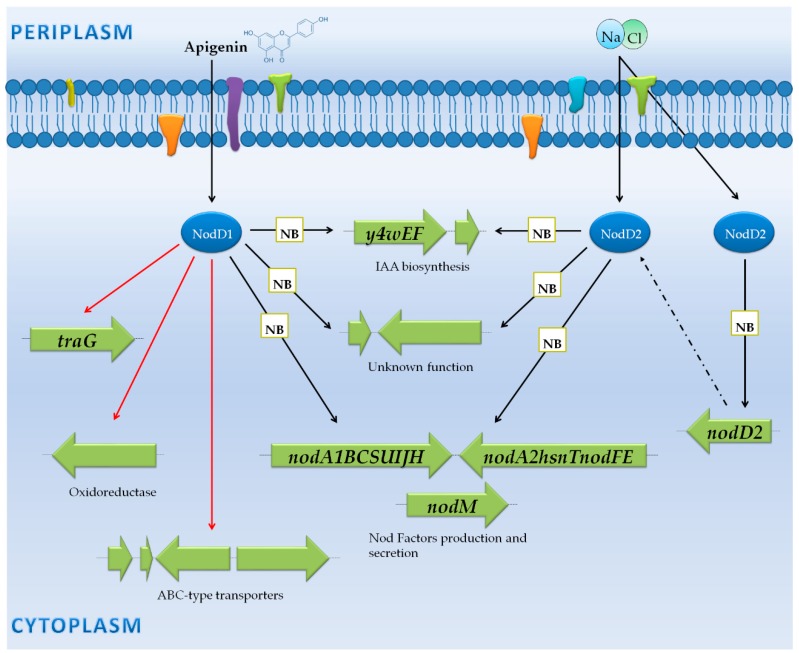
Transcriptomic response in the presence of apigenin and NaCl in *R. tropici* 899. Proteins are illustrated by blue circles. The DNA to protein translation process is represented by dotted arrows. Activation and repression of genes are indicated by black and red arrows, respectively. Genes regulated by a *nod* box are represented in green.

Recently, Pérez-Montaño et al. [44] performed the first transcriptomic study in *R. tropici* CIAT 899 in the presence of apigenin and salt. This work revealed that 19 genes are differentially expressed when the bacterium was grown in the presence of apigenin, being 15 genes upregulated and located on the symbiotic plasmid, meanwhile the other 4 are downregulated and distributed in other replicons. Interestingly, with respect to the cultures supplemented with salt, the same symbiotic-related genes are also upregulated, but many other non-symbiotic genes are also differentially expressed (95 upregulated and 628 downregulated) (Table S3). Interestingly, in a second transcriptomic approach, del Cerro et al. [45] confirmed that the transcriptional activation of previously mentioned genes upon apigenin and salt conditions are mediated by NodD1 and NodD2, respectively. Taking in consideration both transcriptomic studies, two groups of genes are identified: (i) genes controlled by *nod* boxes (NB); and (ii) genes not preceded by a NB (Figure 4).

#### 3.3.1. Genes Controlled by NB

Four sets of genes are activated in the symbiotic plasmid (NC_020061) in a NodD1-apigenin dependent manner and/or in a NodD2-salt dependent manner. Two of them corresponded to the *nodA1BCSUIJHPQ1Q2* and *nodA2hsnTnodFE* operons (NB1 and NB2, respectively), the third is involved in the synthesis of the phytohormone indole-3-acetic acid (IAA) (NB4), and the last set of genes (*PB01550* and *PB01545*) codes for proteins with unknown functions (NB5) [44]. Although *nodM* expression did not reach the threshold considered by the authors of the study (fold change ±4), qRT-PCR (quantitative Real Time-Polymerase Chain Reaction) results indicated that this gene is also significantly expressed in the presence of the flavonoid apigenin [44]. Therefore, at least two main biological processes that are beneficial for symbiosis are activated in this bacterium when induced with apigenin or salt: the synthesis and export of NF, that will induce root nodule primordia formation (*nodA1BCSUIJHPQ1Q2*, *nodA2hsnTnodFE*, and *nodM*) and the production of indole-3-acetic acid (IAA) that will favour root development (*y4wEF*). Interestingly, the PB01055 gene, which is located on the symbiotic plasmid under the control of NB7 and codes for a putative calcium/calmodulin dependent protein kinase II, is only transcriptionally activated in the presence of salt. As commented above, the transcriptomic analyses indicate that NodD1 is not involved in the transcriptional activation of symbiotic genes under salt stress. However, there is an exception, since transcription of the *nodD2* gene is controlled by NB9 in a salt-NodD1 dependent manner. This finding indicates that NodD1 could increase *nodD2* expression and, consequently, the production of NF in the presence of high concentrations of salt (Table S3) (Figure 4).

#### 3.3.2. Genes Not Preceded by a NB

According to the transcriptomic study carried out by Pérez-Montaño et al. [44], 4 genes are differentially repressed in a NB-independent manner in the presence of inducing flavonoids. These genes are located in different replicons and code for conjugal Transfer protein (TraG) (located on the 0.22-Mb pRtrCIAT899a), 2 ABC-type transporters (one on the 3.8-Mb chromosome and the other on the 2.08-Mb pRtrCIAT899c) and an oxidoreductase (located on pRtrCIAT899c). However, so far, no reports have been published describing the implication of any of these genes in the rhizobium–legume symbiosis (Table S3) (Figure 4). Interestingly, it has been recently described that TraG is critical for promoting the transference of conjugant elements from *Azorhizobium caulinodans* to *M. huakuii* in the rhizosphere [100]. Thus, the up-regulation of the *R. tropici* CIAT 899 *traG* gene in response to apigenin might indicate that rhizobia, when sense the presence of a putative host legume plant, could transfer by conjugation its symbiotic plasmid to related bacteria. 

### 3.4. Sinorhizobium Meliloti 1021: Only Production and Export of NF?

*S. meliloti* 1021 is a soil bacterium able to induce the formation of nodules in a very reduced number of legume genera: *Medicago*, *Melilotus* and *Trigonella*, including *Medicago sativa* (alfalfa) and the model legume *Medicago truncatula*. In *S. meliloti*, the expression of the nodulation genes, *nod*, *nol*, and *noe* operons, can be induced by three different NodD proteins: NodD1 and NodD2, which are activated by specific flavonoids and expressed constitutively, and NodD3, whose expression is subject to a complex regulation involving the regulatory proteins SyrM and NodD1, and the nitrogen status of the cell [101,102]. Although inactivation of the three *nodD* genes is required to completely abolish nodulation, the symbiotic relevance of each of these regulators depends on the host legume. Thus, the main regulator for nodulation with *Medicago sativa* is NodD1 [101].

The tripartite genome of *S. meliloti* 1021 comprises a 3.65-Mb chromosome and the 1.35-Mb pSymA and 1.68-Mb pSymB megaplasmids [103]. Many transcriptomics reports relevant to symbiosis have been previously published for this bacterium (see Table 1). In two of them, the transcriptomic responses in the presence of the inducing flavonoid luteolin were assessed [38,46]. In these reports, 24 genes differentially expressed were identified upon flavonoid treatment (Table S4), 18 of them belonging to the *nod*/*noe*/*nol* operons and 6 lacking a NB. However, high variations among replicates were scored by the authors in the expression of several of the genes belonging to the latter group, suggesting that maybe they are not actually differentially expressed genes upon treatment with luteolin. In any case, these transcriptomic studies unequivocally identified two different groups of genes depending on their promoter sequences: (i) genes controlled by NB and (ii) genes not preceded by a NB (Table S4).

#### 3.4.1. Genes Controlled by NB via NodD1

Eighteen upregulated genes controlled by six NB were identified on the pSymA of *S. meliloti* 1021. Five canonical *nod* boxes are located upstream of the *nodABC*, *nodFEGP1P2*, *nodH*, *nodLnoeAB* and *nodMnolFG* operons and another less conserved *nod*-box is situated upstream of *syrM* [46]. The proteins encoded by genes upregulated in the presence of luteolin via NB are implied in the synthesis and export of NF, with the exception of *syrM* and two not previously described genes (SMa0850 and SMa0848). 

#### 3.4.2. Genes Not Preceded by a NB

The absence of *nod* boxes in the promoter regions of the differentially expressed genes SMc03151, SMc03167, SMc03168, SMc03169, and *groEL5* suggests a NodD1-indirect mechanism of activation in the presence of flavonoids. The SyrM protein might be responsible for the activation of this set of genes. However, no *syrM* boxes have been found in the promotor regions of these differentially expressed genes [46]. Independently of their regulation, the functions of the proteins encoded by these genes are currently unknown or poorly investigated, and for this reason, further research is needed to advance in the knowledge about the role of these proteins in the symbiosis between *S. meliloti* 1021 and its legume hosts.

### 3.5. Sinorhizobium Fredii HH103: A Hierarchical Regulatory Cascade Controls NF Production and Export, Biosynthesis of Phytohormones, and Assembly of the T3SS

*Sinorhizobium fredii* is a rhizobial species characterized by its extremely broad host range since it is capable of nodulating more than 100 genera of legumes including plants forming determinate (such as *Glycine max*) and indeterminate nodules (such as *Glycyrrhiza uralensis*) [104,105,106]. Different *S. fredii* strains has been described so far, being the four most studied NGR234, USDA257, CCBAU45436, and HH103. In fact, genomic information is available for all of them [107,108,109,110,111]. An interesting difference between these four strains resides in their symbiotic behaviour with soybean (*G. max*); NGR234 cannot nodulate this legume [104], USDA257 and CCBAU45436 induce the formation of nodules only on wild and non-commercial soybeans [112], but HH103 nodulates effectively both non-commercial and commercial American soybeans [113].

*S. fredii* HH103 strain is one of the most studied rhizobial strains, since its genome has been sequenced and the symbiotic capacity of a large number of mutants affected in Nod-factor production, bacterial surface polysaccharides, and the T3SS have been determined [106,109]. The *S. fredii* HH103 genome is composed of a chromosome and six plasmids. The symbiotic plasmid of HH103 harbours, besides to the structural *nod* genes, diverse symbiotic regulatory genes, including *syrM*, *ttsI*, and two copies of *nodD*. In order to know the global expression response of *S. fredii* HH103 to the presence of flavonoids, Pérez-Montaño et al. [47] performed transcriptomic studies in the presence and absence of genistein, an inducing flavonoid of the *nod* genes in this strain. In addition, *nodD1* and *ttsI* mutants were also included in this study to determine the role of NodD1 and TtsI in the regulation of the HH103 genes differentially expressed by genistein.

**Figure 5:**
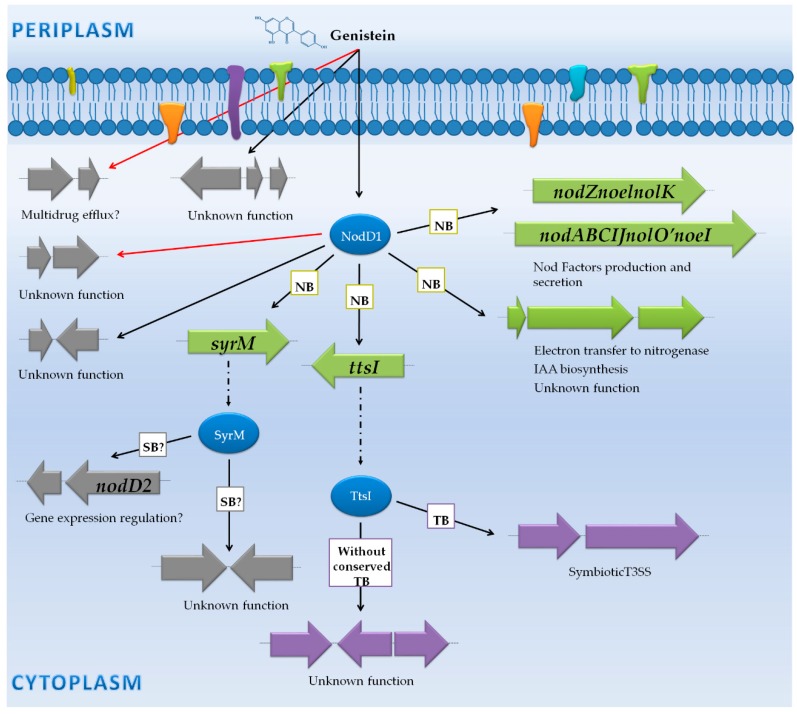
Transcriptomic response in the presence of genistein in *S. fredii* HH103. Proteins are illustrated by blue circles. The DNA to protein translation process is represented by dotted arrows. Activation and repression of genes are indicated by black and red arrows, respectively. Genes regulated by a *nod* box or a *tts* box are represented in green and purple, respectively. Genes which are not controlled by a *nod* box or a *tts* box are indicated in grey. NB: *nod* box; TB: *tts* box; SB?: genes that possibly are controlled by a *syrM* box.

In this transcriptomic report, 100 differentially expressed genes were identified upon genistein treatment (Table S5). Thus, 92 genes were upregulated, most of them located on the symbiotic plasmid (psfHH103d), and only 8 genes were repressed under these conditions. Interestingly, transcriptomic studies carried out with the *nodD1* and *ttsI* mutants unequivocally identified three different groups of genes depending on the presence of functional promoter sequences and on the transcriptional regulator: (i) genes controlled by NB via NodD1; (ii) genes regulated by TB via TtsI; and (iii) genes not preceded by a NB or a TB (Figure 5).

#### 3.5.1. Genes Controlled by NB via NodD1

Fifteen potential NB have been located on the symbiotic plasmid of *S. fredii* HH103 [109]. The transcriptomic comparison between the activation patterns of the wild-type and *nodD1* mutant strains defined 11 of the 15 previously described NB as active (NB1, NB2, NB8, NB9, NB10, NB13, NB14, NB15, NB17, NB18 and NB19). The functions of the proteins encoded by these genes include synthesis and transport of NF (*nodZnoeLnolK* and *nodABCIJnolO’noeI* operons), electron transfer to nitrogenase (*fixABCX* genes), synthesis of the phytohormone IAA (*y4wE* gene), putative production of hopanoids (*hpn* cluster) and transcriptional regulators such as TtsI and SyrM. Interestingly, 4 sets of genes (psfHH103d_116-118, psfHH103d_161, psfHH103d_447-448 and psfHH103d_208), also controlled via NB, NodD1 and flavonoids, code for conserved hypothetical proteins whose functions are currently unknown or poorly investigated [47] (Table S4) (Figure 5). Interestingly, one of these proteins, encoded by psfHH103d_161, could be related to the negative regulation of exopolysaccharide (EPS) production by NodD1 and inducing flavonoids described for *S. fredii* HH103 [31]. This hypothetical protein is 58% identical to *R. leguminosarum* biovar *phaseoli* PsiB, a protein involved in exopolysaccharide inhibition [114]. Further research is needed to clarify this issue. 

#### 3.5.2. Genes Controlled by TB via TtsI

Some Gram-negative bacteria possess a specialized apparatus for protein secretion called T3SS [115]. Some pathogenic and symbiotic bacteria, including rhizobia, use this secretion system to deliver effector proteins into the eukaryotic cell to interfere with signal transduction pathways and promote infection by suppressing defence responses [116]. The transcriptomic study performed in *S. fredii* HH103 corroborated that its symbiotic T3SS is produced and assembled in a flavonoid-, NodD1-, and TtsI-dependent manner, since the *ttsI* gene is located downstream of a functional NB [47]. In addition, this study determined than 11 out of the 18 previously defined as potential TB were functional and controlled the expression of 35 ORFs. All these TB were located on the symbiotic plasmid (psfHH103d) with the exception of TB1 and TB2, present in plasmid psfHH103c. Most of the genes upregulated via TB and TtsI code for components of the T3SS apparatus (*nopBrhcJnolUrhcLNQRSTU* and *nopCAy4yQ*) and other proteins putatively involved in the translocation of proteins to the cytoplasm of the plant cell, such as NopX and GunA. The rest of genes controlled by the remaining TB code for effector proteins, namely *nopC*, *nopD*, *nopI*, *nopM*, *nopM2*, *nopL*, *nopP*, and *nopT* (Table S4) (Figure 5). In fact, many of the proteins encoded by these genes were previously detected in supernatants of *S. fredii* HH103 obtained from cultures induced with genistein [117,118]. These findings indicate that, in the presence of inducing flavonoids, this bacterium produce a fully functional T3SS.

#### 3.5.3. Genes Not Preceded by a NB or a TB

This last group of genes comprises genes differentially expressed in the presence of genistein but not located downstream of a NB or a TB. The transcriptomic analysis indicates that 30 genes distributed throughout the chromosome and plasmids are differentially expressed, 22 of them induced and only 8 repressed when *S. fredii* HH103 is cultured in the presence of genistein (Table S4 and Figure 5) [47]. Among the differentially expressed genes, two operons (psfHH103d_322-psfHH103d_319 and psfHH103d_306-psfHH103_d311), which code for proteins not previously described, seems to be controlled by the regulator SyrM (activated by flavonoids in a NodD1- and NB-dependent manner), since potential *syrM* boxes (SB) has been identified upstream of these genes [47]. On the other hand, seven genes (SFHH103_00346-00348, SFHH103_00844, SFHH103_01920 and psfHH103d_274-275) that also encode for hypothetical proteins, seem to be controlled by both NodD1 and TtsI, but no promoter regions have been identified in the sequences preceding the ORFs. Lastly, the expression of four genes (the SFHH103_03875 gene and the SFHH103_05321-SFHH103_05319 operon) is affected in the presence of genistein in a NodD1- and TtsI-independent manner (Table S4) (Figure 5). The chromosomic SFHH103_03875 gene, which is induced with genistein, codes for a putative membrane permease containing two EamA domains. Despite of this gene is well conserved among different rhizobia, it has not been characterized so far. In contrast, the SFHH103_05321-SFHH103_05319 operon, which codes for a TetR transcriptional regulator and two multidrug efflux transporter proteins, is repressed in the presence of inducing flavonoids. Interestingly, several efflux transporter systems are also differentially expressed in other soybean symbiont, *B. japonicum* USDA 110, in the presence of genistein [43], but further investigations are needed to shed light about the putative role of these transport systems in symbiosis.

## 4. Conclusions

**Figure 6:**
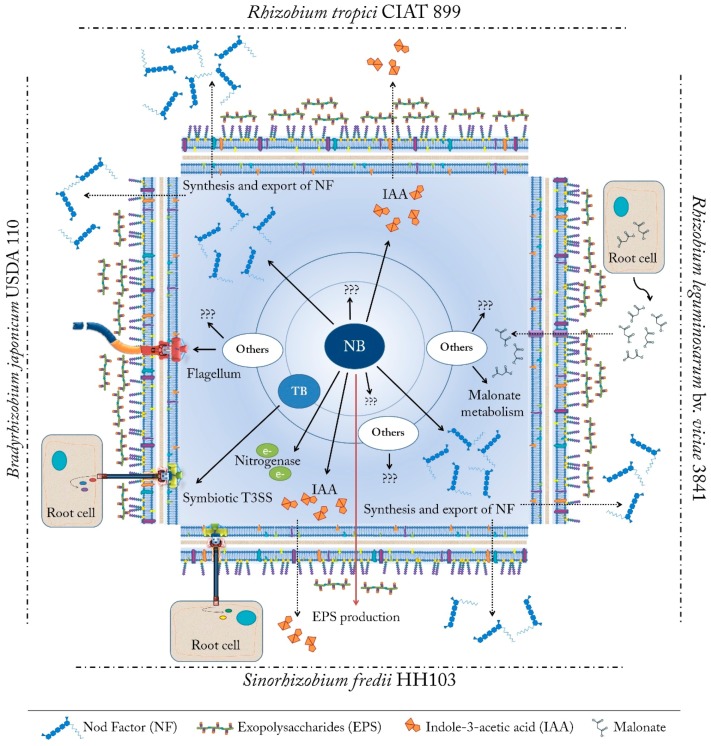
Molecular responses triggered by inducing flavonoids according to transcriptomic reports in several rhizobial strains. NB: *nod* boxes, TB: *tts* boxes. Black arrows: activation. Red Arrows: repression. Dotted arrows indicate transport across the membrane and cell wall.

The understanding of the molecular mechanisms governing the rhizobia–legume symbiosis is crucial for the improvement of this interaction and, consequently, for increasing the yield of crops with high agronomical importance. This process involves an interchange of symbiotic signals between both partners. It is well known that the rhizobial protein NodD is activated by specific flavonoids exuded by legume roots and then binds to conserved promoter sequences activating the transcription of the *nod* genes, which encode for enzymes responsible for the biosynthesis and secretion of the NF. Interestingly, many other symbiotic-related traits are regulated in first instance via flavonoids. In this review, an exhaustive compilation of many symbiosis-related transcriptomic analysis (microarray and RNA-seq) carried out in rhizobia during the last two decades is summarized. Our attention has been focused on the reports describing the transcriptomic changes of several rhizobia triggered upon flavonoid treatment in order to know what are the arsenals of weapons that escorts NF in different rhizobia to successfully infect host legumes. The global transcriptomic reports analysed in this paper indicate that each bacterium has developed specific molecular responses to achieve this purpose (Figure 6). However, the possibility that other processes could be triggered in the presence of inducing flavonoids cannot be ruled out, since the RNA-seq and microarrays techniques commonly give a single frame of the movie. Lastly, further efforts must be performed in order to characterize those differentially expressed genes whose function remains unknown, as well as for the analysis of transcriptomic changes mediated by flavonoids in not previously studied rhizobia. These studies will improve our understanding of the molecular strategies developed by each bacterium to success in the nodulation process and, therefore, shed light on the molecular dialogue established during the symbiotic interaction.

## Figures and Tables

**Table 1:** Summary of the main results obtained in rhizobial transcriptomic studies related with the symbiotic process.

Comparison	Bacteria	Method	Strains	Specific Conditions	Results Related with the Symbiotic Process	References
Free-living cells vs. Bacteroids	*Bradyrhizobium japonicum* USDA 110	Microarray (Affymetrix GeneChip)	Wild-type and RNA polymerase σ^54^ factor (*rpoN1-2)* double mutant	-Free-living cultures in either aerobic or microaerobic conditions (mid-exponential phase). -Bacteroids in soybean (10, 13, 21 and 31 days post inoculation, dpi).	-Distinction among genes expressed in early and late bacteroids. -1/3 of the genes induced in bacteroids (21 dpi) are also upregulated in microaerobiosis, including *fix* and *nif* genes. The other induced genes are regulated by factors other than O_2_ limitation. -Determination of genes transcribed in bacteroids in a σ^54^ (*rpoN1*)-dependent manner (several *fix*, *nif* and *hup* genes included).	[19]
	*B. japonicum* USDA 110	Microarray (Operon Biotechnologies)	Wild-type	-Minimal and rich media (mid-exponential phase). -Osmotic stress conditions (mid-exponential phase). -Bacteroids in soybean (28 dpi).	-15% of the genes of the genome were differentially expressed in bacteroids: N_2_ fixation (*nif* and *fix*) and H_2_ uptake (*hup*) genes up-regulated. Nodulation (*nod*) genes also up-regulated. T3SS (*hrc*) genes are repressed.	[33]
	*Rhizobium leguminosarum* biovar *viciae* 3841	Microarray (Operon Biotechnologies)	Wild-type	-Several carbon substrates (glucose, pyruvate, succinate, inositol, acetate, and acetoacetate). -Bacteroids in vetch (28 dpi). -Bacteroids in pea (7, 15, 21 and 28 dpi).	-386 genes were differentially expressed in at least one stage of bacteroid development. -The decarboxylating arm of the tricarboxylic acid cycle and the aminobutyrate metabolism were highly induced in early nodule bacteroids (7 dpi). -Changes in the expression of regulators, exported and cell surface molecules, multidrug exporters, and heat and cold shock proteins in bacteroids of early pea nodules (7 dpi). -The *fix* genes were induced early but continued to increase in mature bacteroids (15 and 21 dpi), while *nif* genes were induced strongly in older bacteroids of pea nodules (28 dpi).	[34]
	*Mesorhizobium huakuii* 7653R	RNA-Seq Microarray (Roche NimbleGen)	Wild-type	-Bacteroids in *Astragalus sinicus* (32 dpi).	-Mostly, bacteroid up-regulated genes are located on plasmid while downregulated genes are chromosomal. -Free-living cells have a primary role in maintaining basal metabolism, whereas bacteroids in N_2_ fixation (*nif*, *fix* and *suf* clusters up-regulated). -Up-regulation of *rpoN* (σ^54^) and *rpoH* (RNA polymerase σ^32^ factor) indicates an essential role of both transcriptional factors in the activation of genes implied in the N_2_ fixation.	[35]
	*Mesorhizobium loti* MAFF303099	Microarray (Made by Authors)	Wild-type	-Free-living cultures in either aerobic or microaerobic conditions (mid-exponential phase). -Bacteroids in *Lotus japonicus* (42 dpi). -C-starving conditions.	-Clusters of genes within the symbiotic islands are collectively up-regulated in bacteroids (including *nif*, *fix*, *fdx*, and *rpoN* genes) through σ^54^ (*rpoN*). -Most of the upregulated genes in bacteroids are induced in a NifA-independent manner. -Transcription outside the symbiotic island is strongly repressed in bacteroids (genes implied in cell division, cell wall and flagella synthesis).	[20]
	*Sinorhizobium meliloti* 1021	Macroarrays (Made by authors)	Wild-type and *bacA* mutant	-Free-living cultures (minimal and rich media) with and without luteolin. -Free-living cultures in either aerobic or microaerobic conditions. -Bacteroids in young (8 dpi) nodules and in mature nitrogen-fixing nodules (18 dpi) in *Medicago truncatula*. -Bacteroids in *Medicago sativa* mature nodules (18 dpi).	-15 out of 214 tested genes were induced in young and mature nodules of the two strains (specific of the infection stage), including genes that code for hemolysin calcium-binding proteins, adenylate cyclases and type-IV secretion system proteins. -24 out of 214 tested genes were up-regulated in mature nodules of both plants (specific of symbiosis), including *nif, fix* and *cya* genes. -18 out of 214 tested genes were down-regulated in mature nodules of both plants (specific of symbiosis), including genes implied in cell division (*ftsZ*) and chaperonins.	[36]
	*S. meliloti* 1021	Microarray and Macroarray (Made by authors)	Wild-type	-Free-living cultures in either aerobic or microaerobic conditions. -Bacteroids from *M. sativa* nodules (18 to 22 dpi).	-982 genes differentially expressed in bacteroids. -Genes downregulated were implied in cell division (*ftsZ*), DNA, RNA and protein metabolisms (*dna* and *rpo* cluster genes), motility and chemotaxis (*che* genes), phosphorus uptake and utilization (*pho* genes), nitrogen assimilation (*gln* genes) and glucolysis and aerobic respiration (*nuoACEFHIKLMN* operon). -Genes upregulated included mainly those related with symbiosis (several *nod* and *noe* genes), nitrogen fixation (*nif* and *fix* operons) and transport of peptides and aminoacids.	[18]
	*S. meliloti* 1021	Microarray (Made by authors)	Wild-type and *bacA* mutant	-Free-living cultures in early and late exponential and stationary phase. -Bacteroids from *M. sativa* nodules at 5, 8, 14–18 dpi in the wild-type. -Bacteroids from *M. sativa* nodules at 11 dpi in the mutant.	-36 genes were specifically induced in early stages of the symbiosis (*nod* genes and genes implied in the synthesis of the cytochrome c). -Bacterial transcriptomic profile changes during nodule development. -The *nif* and *fix* cluster genes were up-regulated even in young nodules.	[37]
	*S. meliloti* 1021	Microarray (Affymetrix GeneChip)	Wild-type, triple *nodD1 nodD2 nodD3* mutant, *rpoN* and *fixJ* mutants, and triple *nodD1 nodD2 nodD3* mutant overexpressing either *nodD1 or nodD3*	-Minimal and rich media (mid-exponential phase). -Late exponential cultures diluted to 0.15–0.2 OD_600_ and induced for 4 h with luteolin. -Bacteroids from *M. truncatula* nodules at 33–35 dpi.	-RpoN controlled genes that are not differentially expressed in free-living bacteria (*fdx*, *nif* and *fix* operons). -Study of gene expression simultaneously in both symbiotic partners indicate that in total more than 5000 genes are differentially expressed in both organisms. -Most of the plant genes upregulated in wild-type nodules were also induced in Fix^−^ nodules (*fixJ* mutant), indicating that the nitrogen fixing status does not significantly affect plant transcriptomic changes.	[38]
	*S. meliloti* 1021	Microarray (Made by authors)	Wild-type and and *fixJ*, *nifA*, *fixK*, and *nifH* mutants	-Free-living cultures in either aerobic or microaerobic conditions. -Bacteroids from *M. truncatula* nodules at 14 dpi.	-122 genes were activated by FixJ via NifA and FixK (97% located in the symbiotic plasmid), including *fix* and *nif* genes. -FixJ controls the majority of genes expressed in mature bacteroids. -NifA activated genes implied in nitrogen fixation and exopolysaccharides (EPS) production and FixK up-regulated genes involved in respiration, arginine metabolism, denitrification and stress responses.	[39]
	*S. meliloti* 2011	RNA-seq (Illumina Hiseq 2000)	Wild-type and *rpoE2* mutant	-Free-living cultures in mid-exponential or early stationary phase. -Bacteroids from *M. truncatula* nodules at 10 dpi.	-Authors designed EuGene-P, a tool that enables the automated prediction of coding sequences, untranslated regions, transcription start sites and non-coding RNA genes. -Prediction of 6308 coding sequences, 1876 non-coding RNAs and 4077 transcription start sites using cDNA of all conditions.	[10]
	*S. meliloti* 2011	RNA-seq (Illumina Hiseq 2000)	Wild-type	-Bacteroids from *M. truncatula* nodules at 10 and 15 dpi.	-Coupling bacterial and plant gene transcriptome determination to laser dissection, authors determined expression changes at the tissue level on indeterminate nodules.	[40]
	*Sinorhizobium fredii* NGR234	RNA-seq (Illumina Hiseq 2000)	Wild-type	-Bacteroids from *Vigna unguiculata* nodules (21 dpi nodules) -Bacteroids from *Leucaena leucocephala* (35 dpi nodules).	-3143 genes differentially expressed in bacteroids of *V. unguiculata* and 2780 in bacteroids of *L. leucocephala*. -Upregulated genes in bacteroids from both hosts were implied in the synthesis of ATP-binding cassette (ABC) transporters, type 3 secretion system (T3SS), nitrogen metabolism, nitrogen fixation (*nif* and *fix* operons), fatty acid metabolism, benzoate degradation and exopolysaccharide biosynthesis (*exo* cluster genes). -Downregulated genes in bacteroids of both plants were involved in synthesis of DNA, RNA and protein metabolisms and flagellar assembly.	[41]
Aerobiosis (Free-living cells) vs. Microaerobiosis	*B. japonicum* USDA 110	Microarray (Affymetrix GeneChip)	Wild-type and *rpoN1-2* double mutant	-Free-living cultures in either aerobic or microaerobic conditions. -Bacteroids in soybean (10, 13, 21 and 31 dpi).	-Microaerobiosis triggered upregulation of symbiotic relevant genes, including *fix* and *nif* clusters, mostly in a σ^54^-dependent manner. -1/3 of the genes induced in bacteroids (21 dpi) are also upregulated in microaerobic conditions.	[19]
	*M. loti* MAFF303099	Microarray (Made by authors)	Wild-type	-Free-living cultures in either aerobic or microaerobic conditions. -Bacteroids in *L. japonicus* MG20 (42 dpi). -C-starved cells.	-The genome region containing the *fixNOPQ* genes (outside the symbiosis island) and the *fix* and *nif* regions (in the symbiotic island) were upregulated as under both microaerobic and symbiotic conditions.	[20]
	*S. meliloti* 021	Macroarrays (Made by authors)	Wild-type and *bacA* mutant	-Free-living cultures (minimal and rich media) with and without luteolin. -Free-living cultures in either aerobic or microaerobic conditions. -Bacteroids in young (8 dpi) nodules and in mature nitrogen-fixing nodules (18 dpi) in *M. truncatula* J6. -Bacteroids in *M. sativa* mature nodules (18 dpi).	-8 out of 214 tested genes were found to be induced under microoxic and bacteroids conditions, including *fix* and *nif* genes.	[36]
	*S. meliloti* 1021	Microarray (Made by authors)	Wild-type and and *fixJ*, *nifA*, *fixK*, and *nifH* mutants	-Free-living cultures in either aerobic or microaerobic conditions (mid-exponential phase). -Bacteroids from *M. truncatula* nodules at 14 dpi.	-FixJ controlled 74% of the genes induced in microaerobiosis and the majority of genes expressed in mature bacteroids (including *fix* and *nif* genes).	[39]
	*S. meliloti* 1021	Microarray and Macroarray (Made by authors)	Wild-type	-Free-living cultures in either aerobic or microaerobic conditions. -Bacteroids from *M. sativa* nodules (18 to 22 dpi).	-377 genes regulated by oxygen concentration (266 induced and 111 repressed in microaerobic conditions). -31 genes were induced both under microoxic and bacteroids conditions, including genes implied in N_2_-fixation (*nif* and *fix* operons), proline metabolism and denitrification.	[18]
Free-living cells vs. Rhizosphere	*R. leguminosarum* biovar *viciae* 3841	Microarray (Operon Biotechnologies)	Wild-type and mutants in many different genes	-Free-living cells vs. rhizosphere (pea, alfalfa or sugar beet) attached bacteria (7 dpi). -Free-living cultures with and without root exudates (pea). -Free-living cultures with and without the flavonoid hesperetin.	-A common core of 106 genes were rhizosphere-induced genes (70 genes encode for proteins with unknown functions). -The increase of gene expression of the glyoxylate cycle only occurred in the pea rhizosphere. -Many genes on pRL8 (plasmid 8 of *R. leguminosarum*) were specifically up-regulated in the pea rhizosphere.	[42]
Non-induced (Free-living cells) vs. root secretions	*R. leguminosarum* biovar *viciae* 3841	Microarray (Operon Biotechnologies)	Wild-type and mutants in many different genes	-Free living cells vs. rhizosphere (pea, alfalfa or sugar beet) attached bacteria (7 dpi).-Free-living cultures with and without root exudates (pea). -Free-living cultures with and without the flavonoid hesperetin.	-21 genes up-regulated including the nodulation (*nod*) and *rhi* gene clusters on pRL10 (the symbiotic plasmid).	[42]
Non-induced (Free-living cells) vs. flavonoids	*B. japonicum* USDA 110	Microarray (Affymetrix GeneChip)	Wild-type, *nodW* mutant and *nodW* mutant overexpressing *nwsB*	-Free-living cultures with and without genistein (8 hpi).	-101 genes up-regulated in the presence of genistein, including *nod* genes, the flagellar cluster and transport genes. -NodW was essencial for induction of most of these genes. -The phenotype and the gene expression levels in the *nodW* mutant were partially restored by overexpression of *nwsB* gene.	[43]
	*R. leguminosarum* biovar *viciae* 3841	Microarray (Operon Biotechnologies)	Wild-type and mutants in many different genes	-Free-living cells vs. rhizosphere (pea, alfalfa or sugar beet) attached bacteria (7 dpi). -Free-living cultures with and without root exudates (pea). -Free-living cultures with and without the flavonoid hesperetin.	-27 genes up-regulated in the presence of flavonoids, including the *nod* and *rhi* gene clusters on pRL10 (the symbiotic plasmid). -6 genes down-regulated, including *flaD*.	[42]
	*Rhizobium tropici* CIAT 899	RNA-seq (Illumina Hiseq 2000)	Wild-type, *nodD1* mutant and *nodD2* mutant	-Free-living cultures with and without the flavonoid apigenin. -Free-living cultures with and without salt.	-13 symbiotic-related genes up-regulated in the presence of apigenin, including *nod* genes and the IAA synthesis genes. -2 hypothetical-protein genes putatively related with symbiosis were up-regulated via NodD1 and apigenin. -NodD1 activated the expression of the 13 symbiotic-related found genes.	[44,45]
	*S. meliloti* 1021	Macroarrays (Made by authors)	Wild-type and *bacA* mutant	-Free-living cultures (minimal and rich media) with and without the flavonoid luteolin. -Aerobic and microaerobic conditions. -Bacteroids in young (8 dpi) nodules and in mature nitrogen-fixing nodules (18 dpi) in *M. truncatula* J6. -Bacteroids in *M. sativa* mature nodules (18 dpi).	-7 out of the 214 tested genes were induced with luteolin, including the *nod* genes, *traA* and three genes related to iron metabolism (also in nodules).	[36]
	*S. meliloti* 1021	Microarray (Made by authors)	Wild-type and wild-type overexpressing *nodD1*	-Late exponential cultures diluted to 0.15–0.2 OD_600_ and induced for 4 and 24 h with luteolin.	-26 and 5 genes after 4 and 24 h, respectively, were affected in the presence of genistein, including those belonging to *nod*/*nol*/*noe* operons. -Other genes encoding for hyphotetical proteins were also identified (varying among replicates).	[46]
	*S. meliloti* 1021	Microarray (Affymetrix GeneChip)	Wild-type, triple *nodD1 nodD2 nodD3* mutant, *rpoN* and *fixJ* mutants, and triple *nodD1 nodD2 nodD3* mutant overexpressing either *nodD1 or nodD3*	-Minimal and rich media (mid-exponential phase). -Late exponential cultures diluted to 0.15–0.2 OD_600_ and induced for 4 h with luteolin.-Bacteroids from *M. truncatula* nodules at 33–35 dpi.	-Luteolin induced significant expression changes in *nod*/*nol*/*noe* operons (12 genes). -Other 12 genes did not show true flavonoid induction but instead vary in expression depending on replicate.	[38]
	*Sinorhizobium fredii* HH103	RNA-seq (Illumina Hiseq 2000)	Wild-type, *nodD1* and *ttsI* mutants	-Free-living cultures (early stationary phase) with and without the flavonoid genistein.	-100 genes were affected in the presence of genistein: 70 genes induced through *nod* boxes (*nod*, *nol* and *noe* genes) and *tts* boxes (T3SS genes) were upregulated. 30 genes not controlled by NB or TB were differentially expressed in the presence of genistein.	[47]
Non-induced (Free-living cells) vs. saline stress (*nod* gene inducing conditions)	*R. tropici* CIAT 899	RNA-seq (Illumina Hiseq 2000)	Wild-type, *nodD1* mutant and *nodD2* mutant	-Free-living cultures with and without the flavonoid apigenin. -Free-living cultures with and without salt.	-17 symbiotic-related genes up-regulated in the presence of salt, including *nod* genes and the IAA synthesis genes. -All the symbiotic-related genes upregulated in presence of apigenin are upregulated in salt conditions as well. -2 hypothetical-protein genes putatively related with symbiosis were upregulated via NodD2 and salt. -In general, higher *nod* gene expression was detected with salt than with apigenin. -NodD2 activated the expression of the 17 symbiotic-related found genes. -NodD1 enhanced the expression of *nodD2* under salt conditions.	[44,45]

RNA-seq: RNA sequencing; OD_600_: optical density at 600 nm, NB: *nod* boxes; TB: *tts* boxes.

## Supplementary Materials

The following are available online at www.mdpi.com/2073-4425/9/1/1/s1. Table S1. *B. japonicum* 110 differentially expressed genes in the presence of genistein. Table S2. *R. leguminosarum* biovar *viciae* 3841 differentially expressed genes in the presence of hesperetin. Table S3. *R. tropici* CIAT 899 differentially expressed genes in the presence of apigenin. Table S4. *S. meliloti* 1021 differentially expressed genes in the presence of luteonin. Table S5. *S. fredii* HH103 differentially expressed genes in the presence of genistein.
